# Fracture Behavior of Mullite Reticulated Porous Ceramics for Porous Media Combustion

**DOI:** 10.3389/fchem.2019.00792

**Published:** 2019-11-19

**Authors:** Xiong Liang, Yawei Li, Liping Pan, Tianbin Zhu, Qinghu Wang, Benwen Li, Christos G. Aneziris

**Affiliations:** ^1^The State Key Laboratory of Refractories and Metallurgy, Wuhan University of Science and Technology, Wuhan, China; ^2^National-provincial Joint Engineering Research Center of High Temperature Materials and Lining Technology, Wuhan, China; ^3^School of Energy and Power Engineering, Institute of Thermal Engineering, Dalian University of Technology, Dalian, China; ^4^Institute for Ceramic, Glass- and Construction Materials, Technical University Bergakademie Freiberg, Freiberg, Germany

**Keywords:** mullite reticulated porous ceramics, X-ray computed tomography, fracture behavior, strengthening mechanism, dense strut

## Abstract

Mullite reticulated porous ceramics (RPC) are one of the key components for porous media burner, the mechanical properties of mullite RPC decided the service life of the burner. However, the irregularities of cellular structure made it difficult to reveal the fracture behavior of mullite RPCs. In this study, the three-dimensional (3-D) structures of mullite RPCs were analyzed by X-ray computed tomography. The strength and damage behavior of mullite RPCs were respectively investigated via the compression tests and finite element modeling based on the actual 3-D model, also the corresponding strengthening mechanism was proposed. The results indicated that the reconstructed 3-D model exhibited the real microstructure of mullite RPCs, containing the hollow struts and strut defects. The Young's modulus calculated from actual 3-D structures was lower than that from Gibson-Ashby theory. In addition, the surface defects preceded triangular tips to generate the area of stress concentration, leading to the fracture behavior first occurred at the strut defects. With the formation of dense strut in mullite RPCs, the stress uniformly distributed in the whole solid skeleton, thus significantly improving the damage resistance of mullite RPCs.

## Introduction

With the increasing of the energy crisis and consciousness of environmental issue, porous media combustion has attracted people's attention due to its low pollutant emission, high powder density and combustion efficiency (Trimis and Durst, 1996; Wood and Harris, 2008; Yu et al., 2013). Because of the heat recirculation from the burned hot downstream gas to the unburned ones within the burners, the premixed gases can be preheated, thereby the superadiabatic flame or excess enthalpy flame will be yielded (Wu et al., 2015). This combustion technique is being widely used in the field of clean recovery of low calorific gas (Mujeebu et al., 2009). Mullite reticulated porous ceramics (RPCs) with their own characteristics of open cells, three-dimensional networks structure, lower thermal expansion coefficient, higher thermal radiation, and economical to produce are considered as one of the most promising porous media for high-temperature burners (Prochazka and Klug, 2010; Maurath and Willenbacher, 2017; Sandoval et al., 2019). As the key component of porous burner, mullite RPCs must subject to severe thermal stress during the switch on/off of the burner (Pickenäcker et al., 1999). Therefore, the improved mechanical performance of the porous media is critical for their applications.

Generally, RPCs are prepared by template replication with ceramic slurry. This method commonly used the polymer sponge as porous template, and coated with a thixotropic ceramic slurry. After sintering, the RPCs had the same morphology to the original sponge, which exhibited the open cell and three-dimensional structure (Brockmeyer et al., 1990). However, with the removal of polymeric sponge at elevated temperature, the strut defects, such as surface cracks, hollow struts, and their inside tricuspid tips will generate in RPCs. These flaws affect the mechanical properties of RPCs, thereby weakening the life of porous burner (Vogt et al., 2010). Several approaches have been proposed to strengthen the porous ceramics. For instance, the coherence ability between ceramic slurries and sponge was improved via pretreatment of mechanical stretching or sodium hydroxide, thereby increasing the weight of the coated slurry (Yao et al., 2005). Furthermore, the recoating process was developed to eliminate the surface defects of RPCs, and correspondingly thicken the ceramic skeleton (Pu et al., 2004). The novel template with carbon black slurry coated sponge was used to reduce the stress concentration within the hollow struts of RPCs (Jun et al., 2006). Besides, the liquid silicon was immersed into the SiC skeleton to fill up the hollow strut (Fuessel et al., 2011; Ortona et al., 2012). Although the above methods on increasing the strength of RPCs, their fracture behavior and strengthening mechanism are far from being well-understood. The irregular pores and strut defects within the hollow struts lead to the low elastic limit of RPCs because the cracks will appear before the detectable elastic deformation, which are failed to assess the fracture mode and the elastic modulus of the RPCs using the experimental methods (Oliveira et al., 2006; D'Angelo et al., 2012).

Up till now, plenty of simulation approaches are performed to reveal the elastic properties of RPCs. In general, the space-filling polyhedron models including cubic model, Kelvin model and Weaire-Phelan model are widely used for the simulation of porous foams (Gibson and Ashby, 1997; Zhu et al., 1997; Buffel et al., 2014). These models are composed of regular polyhedrons, which are constructed with classic beam shell theory. Meanwhile, the cell struts with circular, triangular, and plateau border cross sectional shapes of Kelvin models were modified to simulate the real structure of foams (Gong et al., 2005; Jang et al., 2008). However, the microstructures of porous ceramics are much more complex than those of the above models, such as the varied pore morphology, pore size, and strut thickness. In order to increase the akin to the real foams, the tessellation-based models including voronoi tessellation and Laguerre tessellation models are built. Tessellation-based models are capable of incorporating foam microstructural variability and irregularity, such as different cell size, strut thickness variation, strut curvature (Song et al., 2010; Colloca et al., 2012; Chen et al., 2015). However, the microstructural characteristics, like the internal strut micromorphology of hollow struts with triangular voids, surface crack, and defects in RPCs are still too complicated for tessellations to construct.

It is well-recognized that the global properties of RPCs are dependent on the reticulated structure and strut microstructure of RPCs (Rezaei et al., 2014). The model construction based on the real ceramic struts is the critical to obtain the elastic properties of RPCs. X-ray computed tomography (CT) is usually used to *in-situ* observe the microstructure inside the materials, which is an effective technique to reconstruct the complicated morphology of porous ceramics (Fischer et al., 2009; Ortona et al., 2010; Chen et al., 2017). In this work, the mullite RPCs with two kinds of strut structures of hollow struts and dense struts are fabricated via polymer sponge replica technique as well as vacuum infiltration of ceramic slurry. The strut microstructure of mullite RPCs were analyzed by means of scanning electron microscope and their 3-D structures were reconstructed via X-ray computed tomography. The effects of strut structures and morphological features on the mechanical properties and fracture behavior of mullite RPCs were investigated based on the compression tests and finite element method. Furthermore, the strengthening mechanism for mullite RPCs are revealed, also the corresponding approaches are proposed.

## Experimental Procedure

### Preparation of Mullite RPCs

The commercial available mullite powder (325 mesh, Jiangsu Jinxing Co., Ltd., China) was used as the main raw material. Andalusite (<7.9 μm, Y60, Imerys) and α-Al_2_O_3_ (<2.0 μm, Kaifeng Special Refractories Co., Ltd., China) were the sintering aids. The additives used for preparing mullite slurry were polycarboxylate, sodium carboxymethyl-cellulose, contraspum K1012, and ammonium lignosulfonate, whose detailed manufactures were same to the previous paper (Liang et al., 2019). The above additives were firstly dissolved in deionized water to prepare solution. The mixture powders containing 80 wt% mullite, 15.2 wt% andalusite, and 4.8 wt% α-Al_2_O_3_ were subsequently added into the solution and stirred for 30 min to produce mullite slurry with solid content of 78 wt%.

In this experiment, mullite RPCs were fabricated via the sponge replica method. Polyurethane sponge (10 pores per inch, 50 × 50 × 20 mm^3^, F. M Co. Ltd., Germany) was used as the porous template, then it was coated with the as-prepared mullite slurry via impregnation process. The coated polyurethane sponges were dried at room temperature for 24 h, subsequently treated at 1,500°C for 3 h to prepare mullite RPCs with hollow struts, which named as sample MH.

Furthermore, in order to obtain mullite RPCs with dense struts, the coated template was first pre-fired at 1,300°C to burn out polymer sponges, thereby producing mullite preforms. Secondly, the infiltration slurry used for the vacuum infiltration process was prepared. The additives of dispersant agent (polycarboxylate, BASF Group, Germany) and antifoam agent (contraspum K1012, Zschimmer & Schwarz, Germany) were dissolved in deionized water by stirring for 5 min. The starting materials of mullite powder, α-Al_2_O_3_, and andalusite powder were mixed with the solution. After ball-milling for 3 h, the infiltration slurry was obtained. Finally, the mullite preforms were treated by vacuum infiltration using the same method given in Liang et al. (2016). The as-infiltrated mullite preforms were, respectively, sintered at 1,500°C for 3 h before cooling to room temperature. The sintered mullite RPC were marked as sample MD.

### RPCs Characterization

The bulk density of mullite RPC (ρ_b_) was calculated by the ratio of mass to the volume of the whole sample. In order to analyze the changes of strut diameter in mullite RPC before and after vacuum infiltration, the macro image of RPC was taken by a digital camera, and the strut diameter was measured by the means of Image-Pro Plus software (Media Cybernetics, Inc., Netherlands). The apparent density (ρ_s_) of mullite struts were tested using mercury porosimeter (AutoporeIV9500, Micromeritics Instrument Corp., USA). Meanwhile, the relative density of mullite RPC was the ratio of bulk density (ρ_b_) to the strut density (ρ_s_). The micromorphology of the sintered struts and strut surface were observed using scanning electron microscope (SEM, Quanta 400, FEI Company, USA). Furthermore, the internal structure of mullite RPC was analyzed via the scanning of X-ray computer tomography (μ-CT, d2, diondo GmbH, Germany). The device was equipped with a 160 KV X-ray source and a Dexela detector 1512 (PerkinElmer, Germany) with a resolution of 1,944 × 1,526 active pixels. In the tomography scanning process, the device was operated at 120 kV and 50 mA, and the reconstructed voxels had a resolution of 25 μm. Based on the obtained tomography image, VG Studio Max 3.0 (Volume Graphics GmbH, Germany) was used as the visualization software to reconstruct the 3D model.

### Mechanical Characterization

The strength of mullite RPC was characterized by the cold crushing strength (CCS), which was tested on the universal testing machine (ETM, Wance, China). The method of CCS test is consistent with the (Goretta et al., 1990), six samples in group were tested to get the average strength to represent the CCS of mullite RPC, also a load speed of 0.5 mm/min was applied during the strength measurement. The Young's modulus of mullite RPCs (rectangular sample of 20 × 20 × 100 mm^3^) were determined by the impulse excitation technique at room temperature (RFDA, Genk, Belgium). The fracture strength of mullite strut was measured as the clod modulus of rupture (CMOR) of the sintered mullite slurries. In addition, the FEA modeling was performed to simulate the effective Young's modulus, Von Mises distribution of the struts during uniaxial compression via ANSYS software.

## Microstructure and Physical Properties of Mullite RPCs

**Figure 1:**
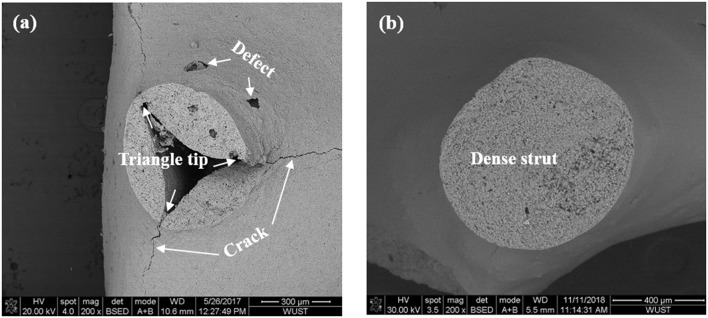
SEM micrographs of the cross section of mullite struts **(A)** Sample MH **(B)** Sample MD.

In order to investigate the strut structure of the prepared mullite RPCs, the micrographs of ruptured struts as well as the strut surface in mullite RPCs are analyzed, shown in Figure 1, respectively. In sample MH, the hollow strut with obvious triangle tips was observed because of the burnt out of polyurethane template (Figure 1A). However, the hollow void within mullite strut was filled up and the triangle tips disappeared in sample MD with the vacuum infiltration of mullite slurry. Meanwhile, mullite skeleton was coated by infiltration slurry, thus forming the dense strut in sample MD (Figure 1B). The coated infiltration slurry on the surface of mullite skeleton could repair the strut cracks and defects in mullite RPCs. It was clearly seen that the large longitudinal cracks and surface defects appeared in sample MH with hollow strut (Figure 1A), while the smooth strut with crack-free showed in sample MD (Figure 1B). Furthermore, the large strut defects were eliminated in the formed dense struts.

The physical properties of mullite RPCs are given in Table 1. It was clearly seen that the strut structure decided the physical properties of mullite RPCs. As for sample MD with dense struts, its bulk density was 0.49 g/cm^3^, which was much larger than that of sample MH. In mullite RPCs with dense struts, the triangular void within hollow strut was completely filled up, and the strut surface was coated by infiltration slurry. Thus, the increased relative density and the strut diameter obtained in sample MD. Furthermore, the dense strut resulted in a higher strength of mullite RPCs. With the formation of dense struts in mullite RPCs, the CCS of mullite RPCs increased from 0.26 to 0.63 MPa.

**Table 1:** Physical properties of mullite RPCs.

**Sample**	**Bulk density (g/cm^**3**^)**	**Skeleton density (g/cm^**3**^)**	**Relative density ρ_**rel**_**	**Strut diameter (mm)**	**CCS (MPa)**
MH	0.28 ± 0.01	1.86	0.15 ± 0.01	0.49 ± 0.07	0.26 ± 0.03
MD	0.49 ± 0.02	2.33	0.21 ± 0.01	0.59 ± 0.01	0.63 ± 0.02

**Figure 2:**
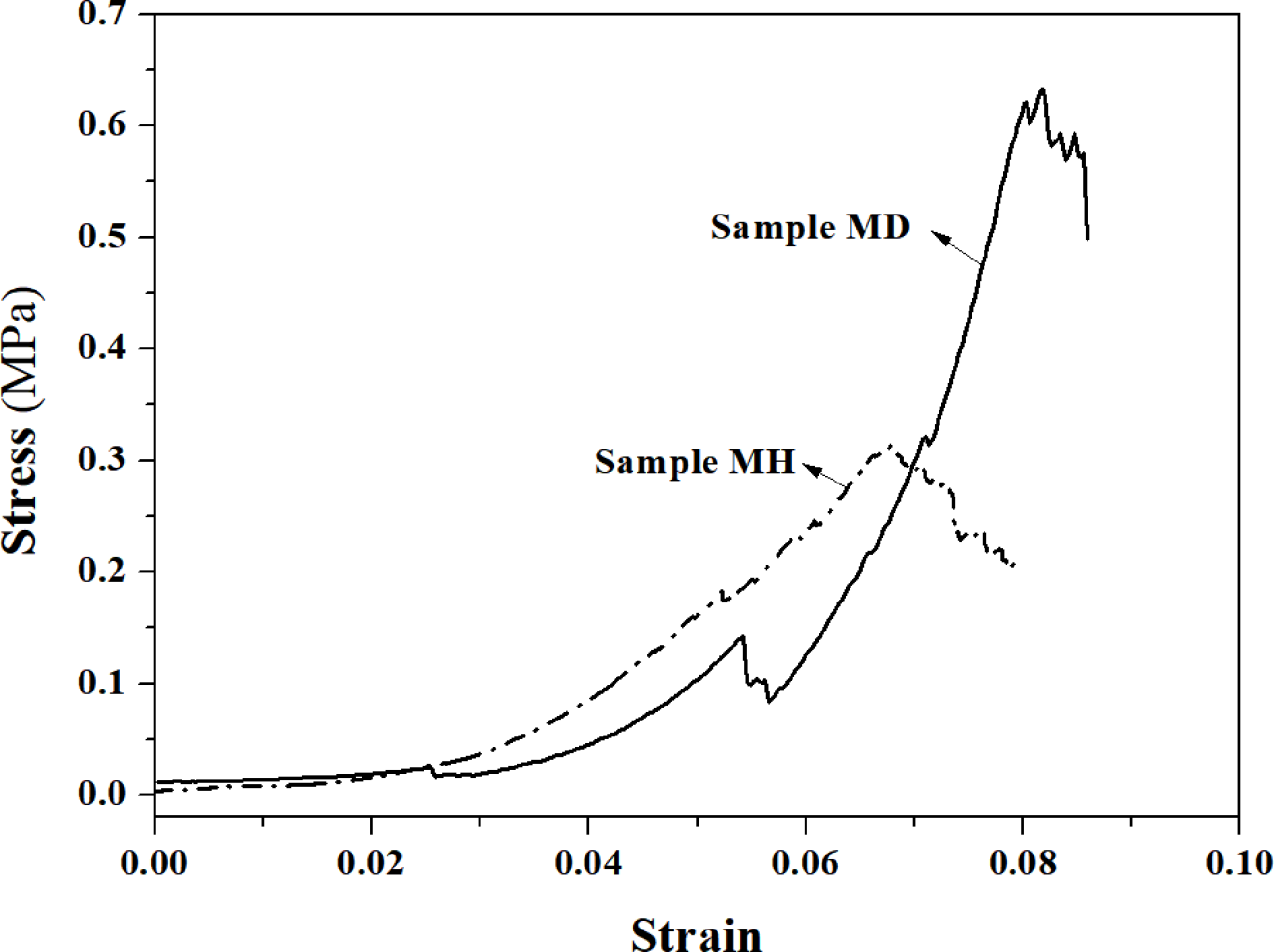
The stress-strain curves of mullite RPCs in the uniaxial compression test.

The stress-strain curves of mullite RPCs under uniaxial compression are shown in Figure 2. It is apparent that the stress of mullite RPCs increased with the strain, whose curves exhibited the zigzag shape. The stress and strain of mullite RPCs with dense struts were larger than that of sample MH. It was noteworthy that the strains of mullite RPCs were both smaller than 0.08.

## Modeling of the Mechanical Properties

### X-ray Computed Tomography

**Figure 3:**
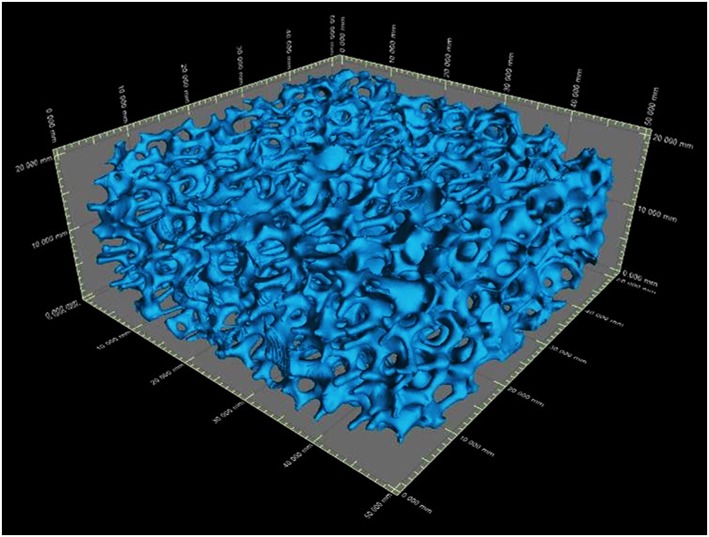
The reconstruction image of the 3-D structure of mullite RPCs.

**Figure 4:**
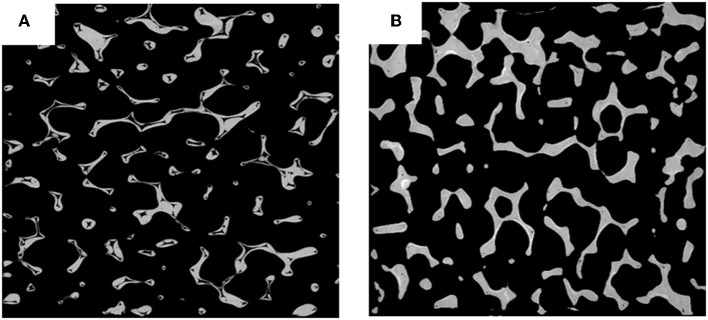
The slice image of the 3-D structure of mullite RPCs **(A)** MH, **(B)** MD.

The 3-D reconstructed structures of mullite RPCs consisted of open-cells skeleton and unregular struts are shown in Figure 3. The reconstructed structures could truly characterize the microstructure of mullite RPCs. In the reconstructed X-ray CT slice of sample MH, the hollow struts with surface defects were observed in Figure 4A. In the reconstructed structure of sample MD, the surface defects and triangular voids within the struts disappeared and dense struts formed (Figure 4B), which was same to the microstructure of mullite RPCs (Figure 1). Furthermore, compared with the slice images of sample MH and MD, it could be seen that the strut diameter of sample MD was larger than that of sample MH. The results exhibited the similar trend to the measured ones (Table 1).

### Model Reconstruction

**Figure 5:**
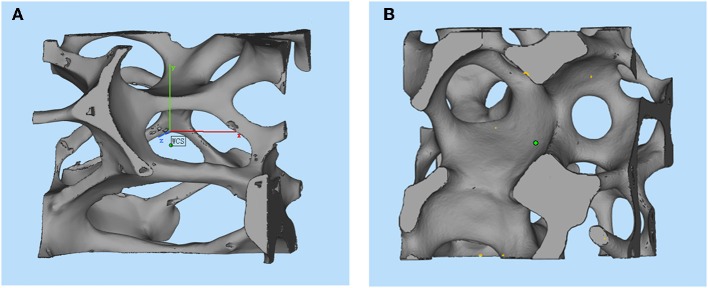
Reduced 3-D structure of mullite RPCs for finite element modeling **(A)** MH, **(B)** MD.

The mechanical properties of mullite RPCs were modeled using the finite element analysis based on the reconstructed models. In order to reduce the computing time, the typical model with the dimension of 8.8 × 8.8 × 8.8 mm^3^ was chosen, showed in Figure 5. The effective physical parameters of the solid struts in mullite RPCs were characterized using the slip casted mullite slurry. After firing at 1,500°C, the Young's modulus (*E*) and Poisson ratio (γ) of the casted mullite slurry were tested by impulse excitation technique. The strength of mullite strut was measured as the clod modulus of rupture (CMOR) of sintered mullite slurries. From the experimental results, the value of *E* and γ for the simulation of reconstructed models were tested as 38.12 GPa and 0.27, respectively. The fracture strength of solid strut was 38.6 MPa (Table 2). Furthermore, the Young's modulus of mullite RPCs was much lower than that of mullite slurry because of the high porous structure. It was worth noting that the elimination of strut defects was beneficial for the improvement of Young's modulus in mullite RPCs. The Young's modulus of sample MH was 0.10 GPa, while the value increased to 2.26 GPa in sample MD.

**Table 2:** Physical parameters of mullite slurry and mullite RPC after firing at 1,500°C.

	**Mullite slurry**	**Mullite RPC**	
		**Sample MH**	**Sample MD**
Young's modulus (*E*, GPa)	38.12	0.10	2.26
Poisson ratio (γ)	0.27	—	—
Flexural strength (CMOR, MPa)	38.6	0.33	0.91

Three deformation steps of mullite RPCs in the process of uniaxial compression were simulated based on the reconstructed models. The deformation was achieved by applying a uniaxial displacement to all the nodes belonging to two parallel faces of the model, and the other faces were fixed. Furthermore, the applied displacements were 5 × 10^−4^, 1.0 × 10^−3^, and 5.0 × 10^−3^ mm, respectively.

### Finite Element Modeling

#### Effective Elastic Modulus

The uniaxial apparent strain along the loading direction was simulated as 0.006, 0.011, and 0.055%, respectively (Table 3). Because the effective Young's modulus of mullite RPCs was determined by the linear elasticity behavior, the smallest strain was chosen to calculate the reactive force in the uniaxial direction when mullite RPCs were under compression. Therefore, the Young's modulus of mullite RPCs were simulated using the actual 3-D models when the strain of 0.006% was applied. The simulated Young's modulus of sample MH and MD were 0.77 and 1.48 GPa, respectively. For comparison, the Young's modulus of the mullite RPCs was also calculated with the Gibson-Ashby model, and the equation was as follows (Fuessel et al., 2011):

$$\begin{array}{l} E=\;C_{1}\cdot E_{\text{S}}{\left(\rho_{\text{rel}}\right)}^{n} \end{array}$$

Where ρ_rel_ was the relative density of RPCs, *E* and *E*_s_ was the effective Young's modulus of mullite RPCs and that for their solid struts (Table 2). Because the RPCs prepared in this work had a complete open cell skeleton, here *n* was chosen as two and *C*_1_ was a proportionality factor close to one (Knackstedt et al., 2005).

**Table 3:** Steps in FEA calculation and calculated reaction force and apparent stress.

**Step**	**Displacement (mm)**	**Strain (%)**	**Model MH**		**Model MD**	
			**Reaction force (N)**	**Apparent stress (MPa)**	**Reaction force (N)**	**Apparent stress (MPa)**
1	0.0005	0.006	3.4	0.044	6.5	0.084
2	0.001	0.011	6.8	0.088	13.0	0.168
3	0.005	0.055	33.8	0.436	65.0	0.839

Table 4 showed the results of effective Young's modulus of mullite RPCs based on Gibson-Ashby model and the actual 3-D structure. It was clearly seen that the Young's modulus calculated by the finite element analysis was lower than that from Gibson-Ashby model. Because the reconstructed models from X-ray tomography exhibited the real microstructure of mullite RPCs, the strut defects and irregular pores were included (Figure 4). However, Gibson and Ashby model consumed that porous materials were ideal matrix, which consisted of the equiaxed open-cell cubic shaped pores.

**Table 4:** The calculated Young's modulus from finite element analysis and Gibson-Ashby theory.

**Model**	**Gibson-Ashby theory (GPa)**	**Finite element analysis (GPa)**
MH	0.86	0.77
MD	1.68	1.48

#### Stress Distribution

The varied strut structures of mullite RPCs led to the difference in stress distribution. In order to reveal the strengthening mechanism of RPCs, the von Mises stress of mullite RPCs with hollow strut and dense strut were calculated during the uniaxial compression. The damage behavior of mullite RPCs was evaluated via the comparison of the von Mises stress to the flexural strength (CMOR) of the solid mullite strut (Table 2). When the von Mises stress was larger than flexural strength, the mullite strut was simulated to be fracture.

**Figure 6:**
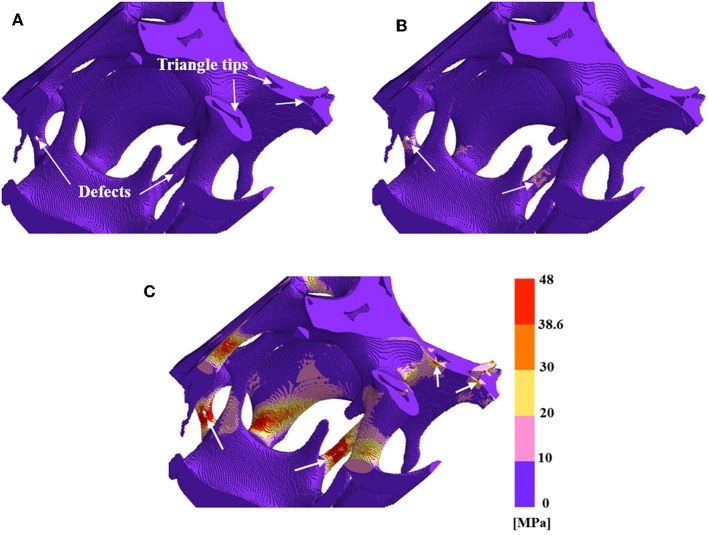
The Von Mises stress and fracture in reduced 3-D structure reconstruction model MH **(A)** at the strain of 0.006%, **(B)** at the strain of 0.011%, and **(C)** at the strain of 0.055%.

Figure 6 shows the von Mises stress of model MH under the applied strains. With the application of a small strain of 0.006%, the stress concentration only appeared at the surface defects of mullite struts, which indicated that the linear elastic behavior occurred (Figure 6A). It verified the validity of the effective Young's modulus calculated by the above finite element method. As the increase of strain to 0.011%, the area of stress concentration enlarged and the value of maximum stress increased correspondingly. Meanwhile, the stress concentration with small areas began to be detected at the triangular tips within the hollow struts (Figure 6B). Under the strain of 0.055%, the large area of damage zone appeared near the strut defects (red regin). Furthermore, the remarkable stress concentration existed at the triangular tips inside the hollow struts (Figure 6C).It was obviously seen that the stress concentration was more likely to form at the surface defects of struts than that at the inner triangular tips in mullite RPCs with hollow struts. In addition, the fracture first occurred at the strut defects during the uniaxial compression. As further increasing the applied strain, the defect-free struts and the struts with larger diameter began to fracture, which resulted in the stress-strain curve with the zigzag shape (Figure 2).

**Figure 7:**
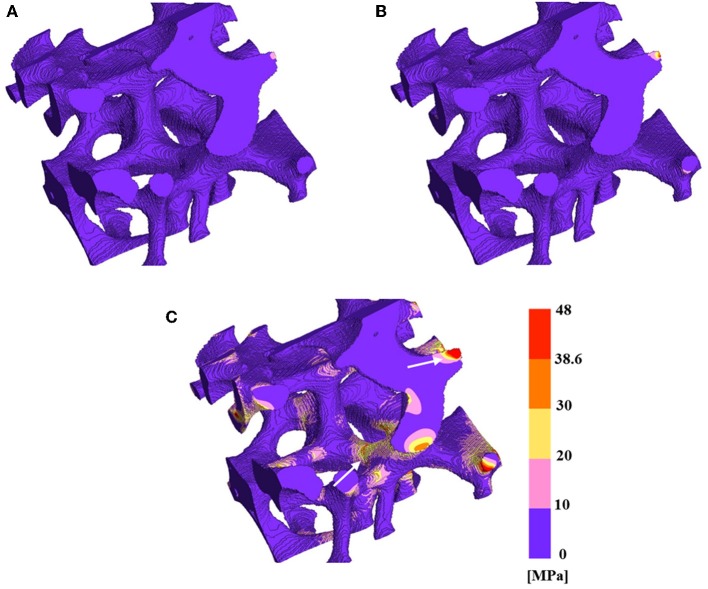
The Von Mises stress and fracture in reduced 3-D structure reconstruction model MD **(A)** at the strain of 0.006%, **(B)** at the strain of 0.011%, and **(C)** at the strain of 0.055%.

The von Mises stress of mullite RPCs with dense struts under the uniaxial compression are presented in Figure 7. It was noteworthy seen that the triangular voids within the struts were completely filled up, also the surface defects disappeared from the reconstructed model MD. When the strain was 0.006%, the stress uniformly distributed in the whole mullite skeleton, and the maximum stress was <10 MPa (Figure 7A). Under the strain of 0.011%, the whole stress was nearly unchanged, only local stress appeared at the strut with small area (Figure 7B). With the strain increasing to 0.055%, the stress increased among the whole struts, especially in the area of finer struts. However, the fracture behavior of mullite RPCs did not occur even under the largest strain (Figure 7C). With the formation of dense struts in mullite RPCs, the stress concentration of mullite RPCs disappeared, thus resulting in the enhanced compression resistance.

It can be seen from Figures 6, 7, the whole structures were not completely failed in model MH and MD when the applied strain was 0.055%. Therefore, the stresses of sample MH and MD could be calculated as 0.42 and 0.81 MPa based on the effective Young's modulus of mullite RPCs from FEA. However, the measured strength of sample MH and MD were 0.29 and 0.63 MPa respectively, which were much lower than the simulated ones. The stress difference between FEA results and experimental tests might be related to the uneven surface of mullite RPCs. During the compression test, the non-uniform loading induced by the rough surface of mullite RPCs caused the stress concentration, thus seriously reducing the strength of RPCs. From the FEA results, the elastic deformation occurred when the strain was much <0.055%. Therefore, the loading displacement should be <1.1 μm during the testing process, which was difficult to be achieved in the actual compression test. The stress concentration induced by the rough surface led to the lower strength of mullite RPCs than the simulation ones, which was unavoidable.

## Conclusions

The strut microstructure and mechanical properties of mullite RPCs were, respectively, investigated via compression tests and finite element analysis based on the reconstructed 3-D models, also the fracture behavior of mullite PRCs was discussed. The following conclusions could be summarized:

1. The reconstructed 3-D models from XCT exhibited the real strut microstructure of mullite RPCs, containing the hollow struts and strut defects. The Young's modulus calculated from the finite element analysis with actual 3-D structures was lower than that from Gibson-Ashby model.
2. The surface defects and triangular tips within hollow struts led to the stress concentration of mullite RPCs during uniaxial compression. The surface defects preceded triangular tips to generate the area of stress concentration, resulting in the fracture behavior first occurred at the strut defects. With the formation of dense strut in mullite RPCs, the hollow strut was completely filled up and the strut defects were eliminated. The dense struts made the stress uniformly distributed in the whole solid skeleton, thus significantly improved the damage resistance of mullite RPCs.

## Data Availability Statement

All datasets generated for this study are included in the article/supplementary material.

## Author Contributions

XL, YL, TZ, and QW contributed conception and design of the study. LP and BL organized and analyzed the database. CA revised the manuscript. All authors contributed to manuscript revision, read, and approved the submitted version.

### Conflict of Interest

The authors declare that the research was conducted in the absence of any commercial or financial relationships that could be construed as a potential conflict of interest.
